# Physiological Response to Facial Expressions in Peripersonal Space Determines Interpersonal Distance in a Social Interaction Context

**DOI:** 10.3389/fpsyg.2018.00657

**Published:** 2018-05-07

**Authors:** Alice Cartaud, Gennaro Ruggiero, Laurent Ott, Tina Iachini, Yann Coello

**Affiliations:** ^1^Cognitive and Affective Sciences Laboratory, CNRS, UMR 9193, SCALab, Université de Lille, Lille, France; ^2^Laboratory of Cognitive Science and Immersive Virtual Reality, CS-IVR, Department of Psychology, University of Campania Luigi Vanvitelli, Vanvitelli, Italy

**Keywords:** peripersonal space, interpersonal space, facial expression, electrodermal activity, physiological response

## Abstract

Accurate control of interpersonal distances in social contexts is an important determinant of effective social interactions. Although comfortable interpersonal distance seems to be dependent on social factors such as the gender, age and activity of the confederates, it also seems to be modulated by the way we represent our peripersonal-action space. To test this hypothesis, the present study investigated the relation between the emotional responses registered through electrodermal activity (EDA) triggered by human-like point-light displays (PLDs) carrying different facial expressions (neutral, angry, happy) when located in the participants peripersonal or extrapersonal space, and the comfort distance with the same PLDs when approaching and crossing the participants fronto-parallel axis on the right or left side. The results show an increase of the phasic EDA for PLDs with angry facial expressions located in the peripersonal space (reachability judgment task), in comparison to the same PLDs located in the extrapersonal space, which was not observed for PLDs with neutral or happy facial expressions. The results also show an increase of the comfort distance for PLDs approaching the participants with an angry facial expression (interpersonal comfort distance judgment task), in comparison to PLDs with happy and neutral ones, which was related to the increase of the physiological response. Overall, the findings indicate that comfort social space can be predicted from the emotional reaction triggered by a confederate when located within the observer’s peripersonal space. This suggests that peripersonal-action space and interpersonal-social space are similarly sensitive to the emotional valence of the confederate, which could reflect a common adaptive mechanism in specifying theses spaces to subtend interactions with both the physical and social environment, but also to ensure body protection from potential threats.

## Introduction

The space around the body is essential to interact physically and socially with the environment. Conceptualized as the peripersonal space, it is conceived as a multisensory interface between the body and the environment where objects can be reached and are naturally coded in terms of potential actions (Rizzolatti et al., 1997; Previc, 1998; Berti and Frassinetti, 2000; Holmes and Spence, 2004; Coello and Delevoye-Turrell, 2007; Cardellicchio et al., 2011; Iachini et al., 2014; Wamain et al., 2016). Dominant theories of spatial cognition consider that the peripersonal space is represented as an action space depending on the spatial properties of the environment and the dynamic characteristics of the body (Cléry et al., 2015; Coello and Iachini, 2016a; di Pellegrino and Làdavas, 2015). As a consequence, modifying arm length in the body schema through tool-use (Cardinali et al., 2012; Bourgeois et al., 2014) or biasing the spatial outcome of manual reaching action (Bourgeois and Coello, 2012), also modifies the representation of the peripersonal space. Likewise, changing the value of objects in the environment through reward expectations also alters the representation of the peripersonal space (Coello et al., in press). Due to its motor nature, increased activation in the sensorimotor brain areas has been reported when manipulable objects are presented in the peripersonal instead of extrapersonal space, even with tasks focusing on perceptual (Culham et al., 2008; Proverbio, 2012; Wamain et al., 2016), semantic (Wamain et al., 2018) or conceptual information about objects (Coventry et al., 2008; Coello and Bonnotte, 2013).

More recently, peripersonal space has also been described as a safety space contributing to protect the body from external threat (Iachini et al., 2014, 2017; Coello and Iachini, 2016a). In agreement with this, it has been reported that the presence of a threatening stimulus near the body alters the representation of the peripersonal space (Graziano and Cooke, 2006; Coello et al., 2012; Valdés-Conroy et al., 2012; Ferri et al., 2015). Likewise, an object of interest that is at hand could be ignored if it assumes a threat value due to the social situation. Consistently, in a monkey study, Fujii et al. (2007) showed that the parietal activity associated with the presence of a manipulable object within peripersonal space significantly reduced when another monkey, with a dominant status, was looking for the same object. This suggests that a manipulable object can be included or not in the peripersonal space depending on its value and the social context, which implies a specific modulation of the neuronal activity in the pre-frontal cortex in relation with the posterior parietal cortex (Fujii et al., 2009).

As a consequence, the peripersonal-safety space may influence the adjustment of interpersonal distances in social contexts (Hall, 1969; Hayduk, 1978; Teneggi et al., 2013; Knapp et al., 2014), suggesting that social and action spaces share common mechanisms (Iachini et al., 2014; Ruggiero et al., 2016). As evidence, Quesque et al. (2017) revealed an increase of the minimum interpersonal comfort distance after using a long tool, a typical enlargement effect known for peripersonal space (Bourgeois et al., 2014). This indicates that the representation of the peripersonal space constrains the spatial dimension of social interactions (but see, Patané et al., 2016). Interpersonal distances can thus be viewed as the physical space between people where social interactions occur on the basis of their emotional and motivational relevance (Lloyd, 2009), but in relation with the representation of self and others’ peripersonal space (Coello and Iachini, 2016a). However, interpersonal distances may diverge from peripersonal space depending on the degree of affiliation with the interlocutor, defined by different variables such as gender, ethnicity, age, and also previous social experience (Leibman, 1970; Tajfel et al., 1971; Iachini et al., 2016). For instance, Iachini et al. (2016) showed that participants select larger comfort distance than reachability distance, in particular female participants when perceiving an approaching male confederate.

Identifying others’ emotional state is an essential aspect of interpersonal social interactions, for which facial expressions may play a crucial role (Darwin, 1872; Ekman and Friesen, 1971; Buck et al., 1972). Indeed, positive facial expressions generally foster approaching behavior whereas negative ones induce avoidance behavior, which means that the size of interpersonal distances perceived as comfortable may depend on the emotional context (Lockard et al., 1977; Ruggiero et al., 2016). In agreement with a link between peripersonal-action and interpersonal-social spaces, invasion of others’ peripersonal space is usually experienced negatively and can cause intense discomfort and anxiety (Horowitz et al., 1964; Hayduk, 1978; Lloyd, 2009). Furthermore, psychological disorders such as social anxiety (Dosey and Meisels, 1969; Brady and Walker, 1978), claustrophobia (Lourenco et al., 2011), borderline personality disorder (Schienle et al., 2015), autistic spectrum disorders (Gessaroli et al., 2013; Perry et al., 2015; Candini et al., 2017), or anorexia (Nandrino et al., 2017) are characterized by a prevalence of enlarged interpersonal distances for comfortable social interactions. In an fMRI study, Kennedy et al. (2009) reported a bilateral activation of the amygdala, a subcortical brain structure known to play a crucial role in emotion regulation, when the experimenter remained in the participants’ peripersonal space during the scan acquisition. Increase of cortisol level and electrodermal activity (EDA) has also been reported in the context of uncomfortable social distances (McBride et al., 1965; Aiello et al., 1977; Evans and Wener, 2007). Complementary evidence linking emotional, social, and spatial processes came from the observation that surgical resection of amygdala associated with temporal tumor surgery produced a severe deficit in the adjustment of interpersonal distances (Kennedy et al., 2009).

Stimuli valence and action system appear thus to contribute to the representation of both the peripersonal-action space and the interpersonal-comfort distance. However, little is known about the link between the body response to the presence of a confederate in the peripersonal space and the interpersonal comfort distance when socially interacting with the confederate. The previous study by Ruggiero et al. (2016) has shown that peripersonal-action space and interpersonal-social space are both sensitive to the emotional valence of a virtual confederate approaching with different facial expressions. Depending on their valence, facial expressions may carry different emotional states and trigger different physiological responses in the observer, which can be detected in the sympathetic nervous system activation associated with the level of physiological arousal (Lang et al., 1993; Boucsein, 2012). Accordingly, physiological responses triggered by a confederate’s facial expression could be modulated by the peripersonal or extrapersonal position of the confederate. Furthermore, the physiological responses triggered by the confederate’s facial expression in peripersonal space could be predictive of the interpersonal comfort distance in a social interaction task. In the present study, we tested these hypotheses by measuring the EDA triggered by a human-like virtual stimulus carrying different facial expressions, and by evaluating whether the interpersonal comfort distance during social interactions can be predicted on the basis of this physiological activity. A reachability judgment task toward the stimuli placed in either the peripersonal or extrapersonal space or at their boundary was used during the EDA recording. Then, a comfort distance judgment task was used to determine the minimum interpersonal comfort distance with stimuli carrying also different facial expressions. We expected that the presence in the peripersonal space of a confederate displaying a negative facial expression should produce a higher EDA in comparison to a confederate displaying a neutral facial expression, more particularly with male confederates who are usually maintained at a larger distance. Moreover, we expected the interpersonal comfort distances to increase in relation to the individual physiological response, in agreement with the protective role of the peripersonal space.

## Materials and Methods

### Participants

Thirty-seven healthy participants (17 women, *M* age = 21.7 years, *SD* age = 2.79) with normal or corrected-to-normal vision participated in the experiment. Participants gave written consent to take part in this study. The protocol received approval by the local Institutional Ethics Committee (Reference No. 2016-2-S41) and conformed to the principles of the Declaration of Helsinki (World Medical Association, 2013).

### Materials and Stimuli

**FIGURE 1:**
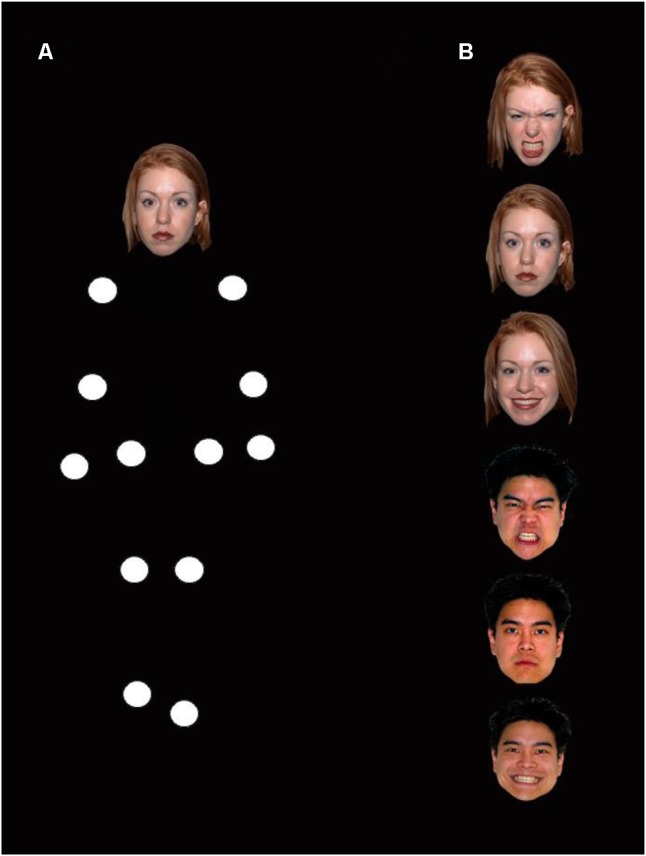
**(A)** Illustration of the PLD used in the experiment (with a neutral facial expression). **(B)** Illustration of the facial expressions used in the experiment.

A schematic representation of the apparatus is presented in **Figure 1A**. Participants were standing at a distance of 1 m from a 4 m × 2 m screen, on which 3D visual stimuli were projected using rear projection from a stereoscopic video projector (Christie Mirage 4K25 DLP 3D projector). The visual stimuli consisted of human-like point like displays and were projected at 120 Hz with a 4 K spatial resolution (3840 × 2060 pixels). Active 3D eyewear (Christie) was used for producing 3D image perception. Stereoscopic images were displayed with off-axis projection by using non-symmetrical camera frustums in order to prevent vertical parallax while providing comfortable stereo pairs. The images were generated according to the participants’ height and inter-pupillary distance. Thus, each eye received a different image for each stimulus alternately displayed at the rate of 8.33 ms. Normal fusion allowed perceiving the 3D moving visual stimuli and distances through relative size and binocular disparity.

The stimuli consisted of human-like point-light displays (PLDs) representing adult males or females oscillating in place or walking toward the participants (Johansson, 1973). The PLDs were generated from adult models captured with a Vicon motion capture system, recording by means of six MX F20 near-infrared cameras (frequency 240 Hz) the position of 39 infrared markers distributed on the body and limbs (see Mouta et al., 2012 for a detailed description). The positions of 13 white dots (54 cd/m^2^) on a black background (0.4 cd/m^2^) were calculated by interpolation from the location of the markers, and signalized the motion of head as well as the left and right ankles, knees, hips, wrists, elbows, and shoulders. Pictures of human faces with different expressions were selected from the NimStim battery (Tottenham et al., 2009) and were associated with the dot representing the head on the PLDs. Geometrical characteristics of the head-picture were computed online to match the distance and size of the PLDs. 72 facial expressions were selected from the NimStim set of facial expressions: 12 female and 12 male faces each associated with a happy, angry, and neutral expression (see **Figure 1B**). For each participant, a set 24 facial expressions was pseudo-randomly selected, including 12 female and 12 male faces each being associated with one single emotion resulting in 8 happy, angry, and neutral expressions. This selection process was used in order to avoid any specific effect of a particular expression associated to a particular face.

**FIGURE 2:**
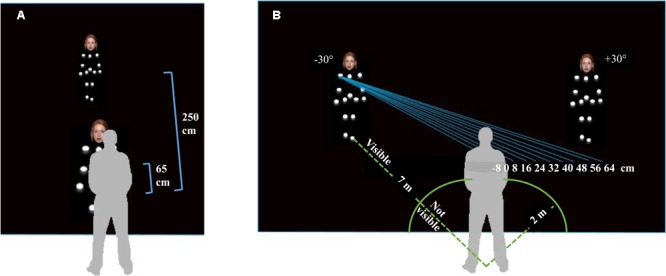
**(A)** Location of the PLD (with a neutral facial expression) when presented in the participants’ peripersonal space (at 65 cm) or extrapersonal space (at 250 cm). PLD located at the boundary of peripersonal space (at 150 cm) is not represented. **(B)** Schematic representation of the within-subjects experimental conditions (not scaled for distance). The PLD started from two different locations (7 m, ±30°), crossed the participants’ mid-sagittal axis, and disappeared at 2 m before virtually passing his/her fronto-parallel plane with an inter-shoulders distance of –8 to 64 cm on the right or left side.

The stimuli were used in two tasks: a reachability judgment task and an interpersonal comfort distance task. In the reachability judgment task, the 24 PLDs with facial expressions were presented in both the participants’ peripersonal space (at 65 cm) and extrapersonal space (at 250 cm, see **Figure 2A**). To allow their perception in 3D, they were oscillating in place without moving their feet. The oscillation activity consisted in a rotation of the whole body around the vertical axis with an angular rotation of about 20 to 30° at a frequency of 0.5 Hz. Another set of 10 PLDs with neutral facial expressions was presented during the reachability judgment task at the boundary of peripersonal space. This boundary was established from a pilot study (*N* = 20) consisting in indicating by pressing on a keyboard key when an approaching PLD (two males, two females, presented twice each) with different facial expressions (angry, neutral, happy) was at a reachable distance (mean: 150 cm, *SD*: 49 cm). In the experiment, the stimuli used were different than the one used in the pilot study and PLDs presented at the boundary of the peripersonal space were essentially used for the purpose of the reachability judgment task.

In the interpersonal comfort distance judgment task (**Figure 2B**), the same set of 24 PLDs with facial expressions were moving toward the participants and the displacement of the PLDs was perceived through the stereoscopic perception of the 13 white dots moving on the black background. In each trial, the PLDs appeared at a distance of 7 m from the participants, walking toward them at a constant speed of 1.2 m/s (simulated looming velocity was constant) and disappeared after having covered a distance of 5 m (thus, at a distance of 2 m from the participants). The PLDs could start walking from a side position located ±30° according to the participants straight ahead (minus sign for left locations). For each starting location, the PLDs could pass the participants’ fronto-parallel plane either on their left or right side. For each side, 10 distances could separate the participants’ and the PLDs’ shoulders at the crossing location, from -8 up to 64 cm by step of 8 cm (negative signs representing collision with the body, see **Figure 2B**). The 0 cm condition was defined according to individual distance between the participants’ mid-sagittal plane and shoulders. Since the PLDs disappeared at 2 m from the participant, the latter had to represent the end of the trajectory mentally until they represent the PLDs passing their fronto-parallel plane.

In the reachability judgment task, physiological responses were registered from EDA through a physiological amplifier BIOPAC MP36 (BIOPAC Systems, Inc., Goleta, CA, United States). Two Ag-AgCl electrodes filled with GEL101 electrolytic mixture were tied on the distal phalanges of the index and major fingers of the non-dominant hand of participants. The temperature of the room during the experiment was maintained at 21°C for all participants and the signal was recorded at a sample rate of 1000 Hz.

### Procedure

Before starting the experiment, the participants were requested to fill a self-administered battery of questionnaires in order to control for exclusion criteria (no recent drug and alcohol consumption or excessive stimulating beverage, no previous history of neurological or psychiatric disorders). They also completed the State-Trait Anxiety Inventory STAI-YB (Spielberger et al., 1983; French version by Bruchon-Schweitzer and Paulhan, 1993) and none of them highlighted depressive symptom (average score for anxiety-state: 31 and anxiety-trait: 41). Then, the experimenter placed the electrodes on the participant’s non-dominant hand and provided instructions concerning the experiment. The participants were placed in front of the vertical screen as described earlier and watched few examples of the human-like PLDs walking toward them from a straight-forward location (0°), and disappearing when reaching the distance of 20 cm from the participants. This practice session was performed in order to familiarize the participants with the virtual environment, the stereoscopic display and the PLDs. It was also performed to assess the correct 3D perception of the stimuli. Then, the participants started with the reachability judgment task and then performed the interpersonal comfort task.

#### Reachability Judgment Task

The reachability judgment task started with a 2 min baseline recording of the EDA while the participants were still staring at a black screen. Then, the reachability judgment task started and the 24 PLDs with different facial expressions were randomly presented in the peripersonal and extrapersonal space (thus 48 stimuli), intertwined with the 10 PLDs with neutral facial expressions presented at the boundary of peripersonal space. Thus, a total of 58 stimuli were randomly presented, articulated in two blocks of trials separated by a rest period. Because we used human-like PLDs, the stimuli were animated with an oscillatory movement so that they were perceptible with a 3D structure. Participants were requested to keep a stable posture and to estimate if the presented PLD was reachable with their dominant hand or not, but without performing the related arm movement. The PLDs were presented for a duration of 6 to 7.5 s (randomly selected), then a question mark appeared on the screen informing the participants that they had to provide their response. Reachable-unreachable responses (i.e., yes–no dichotomous responses) were provided with the index and major fingers of the dominant hand (counterbalanced across participants) using a computer keypad placed on a table located on the participants’ side. A black screen appeared then for a duration of 4 to 5.5 s following the participant’s response.

#### Interpersonal Comfort Distance Judgment Task

Participants had to judge whether the distance at which the PLDs crossed their fronto-parallel plane was comfortable or not (yes–no responses) by pressing one of two keys on the computer keypad with the index and major fingers of their dominant hand (counterbalanced across participants). The PLDs started walking 7 m from the participants, either at +30° or at -30° (for the left side) of eccentricity according to the participants’ straight-ahead. For each starting location, the PLD crossed the participants’ fronto-parallel plane with one of the 10 possible inter-shoulders distance (-8, 0, 8, 16, 24, 32, 40, 48, 56, 64 cm), randomly selected, and disappeared when reaching the distance of 2 m from the participants. The participants provided comfortability judgment after the PLD disappeared and when it had virtually reached the level of their (right or left) shoulder. Thus, 480 trials were performed, divided in three blocks of 160 trials with resting period between the blocks.

### Post-experiment Stimuli Evaluation

Following the experiment, the participants were involved in a post-experiment debriefing and had to evaluate the different facial expressions in terms of emotion (arousal and valence) using the self-assessment manikin (SAM, Bradley and Lang, 1994). The evaluation was presented on a 30″ computer screen using Limesurvey’s software. Overall, the experiment lasted around 2 h.

#### Data Analysis

Participant’s responses and EDA were analyzed using MATLAB R2015b software (MathWorks, Inc., Natick, MA, United States) and statistical analysis was performed using R (version 3.4.1) and R Studio softwares (version 1.0.143). In the *reachability judgment task*, the dichotomic (yes–no) responses were recorded by the computer and the frequency of reachable responses was analyzed through a Space (peripersonal, extrapersonal) × Facial expression (angry, neutral, happy) ANOVA with repeated measures on both factors. The EDA was processed only for the PLDs presented in the peripersonal and extrapersonal spaces. Using the LEDALAB toolbox of MATLAB (version 349, Benedek and Kaernbach, 2010), the physiological signal was down-sampled at 20 Hz and smoothed using the gauss-method with a 32 samples window. We first decomposed the physiological signal into tonic and phasic components using continuous decomposition analysis, then we analyzed the average of the phasic activity over each epoch (CDA.SCR). The time window of interest was 0.5 to 6 s after stimulus onset. Linear mixed-effect model was used to analyze the phasic activity (μS) as a function of Facial expressions (angry, happy, neutral), Space (peripersonal, extrapersonal), PLD Gender (male, female) and Participant Gender (male, female). This data analysis takes into account interpersonal variability as random variables (lme4 1.1-13 package, Bates et al., 2015). According to the full model:

(1) $$\begin{array}{c} Phasic Activity\;=\;(Facial expression * Space + Facial expression + Space + PLD gender + Participant gender +(1 | Participant)) \end{array}$$

Reduced models (i.e., when removing fixed effects of interest) were compared using Likelihood Ratio test distributed like χ^2^ with degrees of freedom corresponding to the parameters estimate of each model. When significant, parameters of the models were associated with the corresponding *t*-value; *p*-values were obtained using normal approximation of the corresponding *t*-values. We also tested the phasic activity as a function of PLDs arousal and valence evaluation (SAM questionnaire). According to the models used:

(2) $$\begin{array}{c} Phasic Activity\;=\;(Arousal * Space +(1 | Participant)) \end{array}$$

(3) $$\begin{array}{c} Phasic Activity\;=\;(Valence * Space +(1 | Participant)) \end{array}$$

Concerning the *comfort judgment task*, the participants’ responses were pooled for PLDs starting from the left and the right position (see Quesque et al., 2017, for details). Perceived minimum interpersonal comfort distance was determined using a maximum likelihood fit based on the second-order derivatives (quasi-Newton method) to obtain the logit regression model that best fitted the comfortable/uncomfortable responses (see Bourgeois and Coello, 2012 for details). We used the equation:

(4) $$\begin{array}{c} y\;=\;e^{(}{{}^{\alpha +}}^{\beta X)}{/(1+e}^{(}{{}^{\alpha +}}^{\beta X}{}^{)}) \end{array}$$

in which y is the participants’ (yes, no) response, X is the crossing distance, and (-α/β) is the critical value of X corresponding to the transition between comfortable and uncomfortable stimuli, thus expressing the perceived minimum comfortable distance. Statistical analyses were carried out using linear mixed-effects model to analyze the variation of minimum comfortable distance (cm) as a function of the condition. According to the full model:

(5) $$\begin{array}{c} Comfort Distance\;=\;(Facial expression + PLD gender + Participant gender +(1 | Participant)) \end{array}$$

We also tested the comfort distance as a function of PLDs arousal and valence evaluation (SAM questionnaire), according to the model:

(6) $$\begin{array}{c} Comfort distance\;=\;(Arousal * Valence +(1 | Participant)) \end{array}$$

With respect to our hypotheses, the relation between the minimum comfort distance (interpersonal comfort distance judgment task) and the EDA (reachability judgment task) was analyzed for the PLDs with different facial expressions when located in the peripersonal space. Then, we used linear mixed-effect models in order to analyze the relation between the EDA phasic activity and the minimum comfort distance, according to the model:

(7) $$\begin{array}{c} Comfort distance\;=\;(Phasic Activity +(1 | Participant)) \end{array}$$

Finally, PLDs arousal and valence evaluations depending on the facial expression (angry, neutral, happy) were analyzed from the SAM questionnaire responses using linear mixed-effects models, as follows:

(8) $$\begin{array}{c} \mathrm{Arousal}\;=\;(Facial expression +(1 | Participant)) \end{array}$$

(9) $$\begin{array}{c} \mathrm{Valence}\;=\;(Facial expression +(1 | Participant)) \end{array}$$

## Results

### PLDs Arousal and Valence Evaluations (SAM Questionnaire)

Concerning arousal evaluation, the value attributed to the PLDs was on average 1.57 (*SD* = 1.20) and depended on the facial expression [χ^2^(2) = 390.31, *p* < 0.001; angry PLDs: 2.23 (*SD* = 1.08); neutral PLDs: 0.47 (*SD* = 0.59); and happy PLDs: 2.01 (*SD* = 0.99)]. The evaluation of angry PLDs differed from the evaluation of happy PLDs (estimate = 1.80, *SE* = 0.08, *t* = 10.2, *p* < 0.001) and neutral PLDs (estimate = 1.94, *SE* = 0.07, *t* = 25.42, *p* < 0.001).

Concerning valence evaluation, the value attributed to the PLDs was on average 1.90 (*SD* = 1.40) and depended on the facial expression [χ^2^(2) = 1195, *p* < 0.001; with for angry PLDs: 0.23 (*SD* = 0.40); neutral PLDs: 1.92 (*SD* = 0.19); and happy PLDs: 3.53 (*SD* = 0.47)]. The evaluation of angry PLDs differed from the evaluation of happy PLDs (estimate = 3.31, *SE* = 0.04, *t* = 78.28, *p* < 0.001), but not neutral PLDs (*t* = 1.2, *p* = 0.22).

### Reachability Judgment Task

Concerning the reachability estimates, PLDs presented in the peripersonal and extrapersonal space were respectively judged as reachable (94.4%) and unreachable (99.10%). Furthermore, reachability judgment for PLDs presented in the peripersonal and extrapersonal space was not influenced by the facial expression [*F*(2,34) = 1.16, *p* = 0.31], and there was no interaction between the two factors [*F*(2,34) = 0.61, *p* = 0.55]. PLDs at the boundary of peripersonal space with neutral facial expression were predominantly judged as unreachable (94.5%).

**FIGURE 3:**
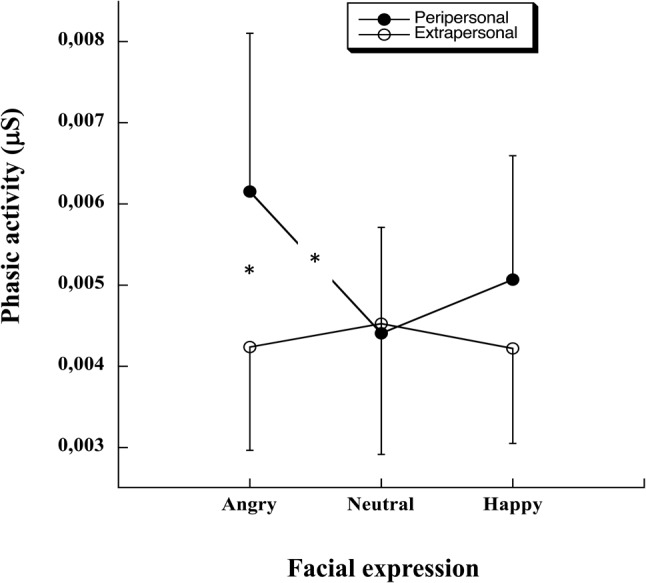
Mean phasic activity (μS) and standard error as a function of the PLDs’ facial expression (angry, neutral, happy) when located in either the participants’ peripersonal or extrapersonal space.

Concerning the EDA phasic activity, statistical analysis revealed a main effect of Space [χ^2^(1) = 7.615, *p* = 0.006] and an interaction between Facial expression and Space [χ^2^(2) = 6.92, *p* = 0.031, see **Figure 3**]. PLDs in the peripersonal space led to an increase of the phasic activity in comparison to PLDs in extrapersonal space (estimate = 0.0006, *SE* = 0.0002, *t* = 2.78, *p* = 0.0054) and the effect was higher for PLDs with angry facial expression than for PLDs with neutral facial expression (estimate = 0.002, *SE* = 0.0006, *t* = 2.95, *p* = 0.0032). Finally, in the peripersonal space PLDs with angry facial expression led to a higher phasic activity in comparison to PLDs with neutral facial expression (estimate = 0.0012, *SE* = 0.0004, *t* = 3.11, *p* = 0.0018). Statistical analysis also revealed an interaction between PLDs arousal evaluation and Space [χ^2^(1) = 7.57, *p* < 0.01]. Stimuli evaluated as highly arousing resulted in a higher phasic activity in the peripersonal space (estimate = 0.0004, *SE* = 0.0002, *t* = 2.01, *p* = 0.045). No other effect was significant.

### Comfort Interpersonal Distance Judgment Task

**FIGURE 4:**
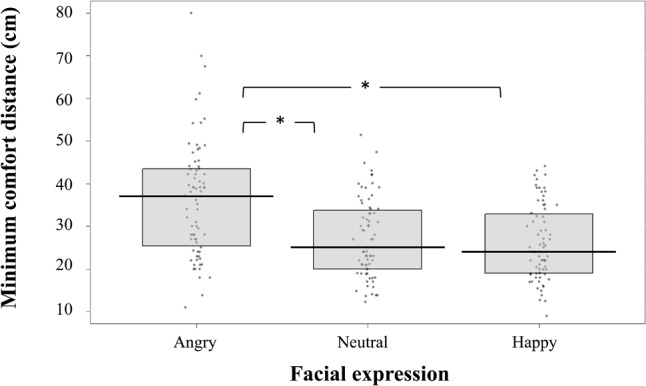
Pirateplot (median and interquartile) representing the variation of minimum comfort distance (cm) as a function of the PLDs’ facial expression (angry, neutral, happy).

Concerning the minimum interpersonal comfort distance (29.70 cm on average), statistical analysis revealed a main effect of Facial expression [χ^2^(2) = 87.15, *p* < 0.01], with an increase of the minimum interpersonal comfort distance for angry facial expressions in comparison to neutral (estimate = 9.29, *SE* = 1.10, *t* = 8.43, *p* < 0.001) and happy facial expressions (estimate = 10.17, *SE* = 1.20, *t* = 8.43, *p* < 0.01, see **Figure 4**). Statistical analysis also showed a main effect of PLDs Arousal evaluation [χ^2^(1) = 73.71, *p* < 0.001] and an interaction between Arousal and Valence [χ^2^(1) = 5.74, *p* = 0.0.02]. PLDs evaluated as highly arousing led to an increase of minimum interpersonal comfort distance (estimate = 3.54, *SE* = 0.70, *p* < 0.001) and the effect was modulated by the valence rating (estimate = -0.76, *SE* = 0.32, *p* = 0.02). No other significant effect was observed.

### Relation Between the EDA Triggered by PLDs in Peripersonal Space and the Interpersonal Comfort Distance

**FIGURE 5:**
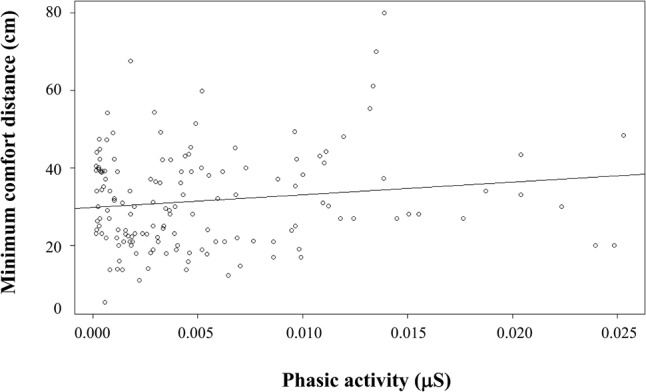
Individual minimum comfort distance (cm) as a function of individual phasic activity (μS) for PLDs with angry and neutral facial expression presented in the peripersonal space. The linear relation indicates that 0.01 μS increase of phasic activity corresponds to an increase of 5.14 cm of minimum comfort distance.

When considering facial expressions producing differences in EDA in the peripersonal space (angry and neutral facial expressions), we observed that the modulation of the phasic activity predicted the modulation of the minimum comfort distance [χ^2^(1) = 7.22, *p* < 0.01], with a gain of 5.14 cm (estimate) per increase of 0.01 μS phasic activity (*SE* = 1.88, *t* = 2.74, *p* < 0.01, see **Figure 5**).

## Discussion

The aim of the present study was to examine how individual physiological response was modulated by human-like stimuli with different facial expressions in the participants’ peripersonal space, and to demonstrate a relation between the individual physiological response and the interpersonal distances felt as comfortable when interacting with the same human-like stimuli. For this purpose, we used a reachability judgment task and an interpersonal comfort distance task, both performed with PLDs displaying happy, angry, or neutral faces.

With respect to the physiological responses in the *reachability judgment task*, we observed that angry, neutral and happy facial expressions triggered different EDAs in the participants. A significant increase of physiological response was registered for PLDs carrying an angry facial expression (arousal: 2.23; valence: 0.23) when located in the participants’ peripersonal space in comparison to participant’s extrapersonal space (gain of 45%) and for those same PLDs in comparison to PLDs carrying a neutral facial expression (arousal: 0.47; valence: 1.92) in participants’ peripersonal space (gain of 40%). These results confirm the protective role of peripersonal space (Kennedy et al., 2009; Coello et al., 2012; Valdés-Conroy et al., 2012; Iachini et al., 2014, 2016; Ruggiero et al., 2016) and suggest that an invasion of the peripersonal space may trigger defensive behavior (Graziano and Cooke, 2006; Cléry et al., 2015; di Pellegrino and Làdavas, 2015). The need of maintaining a safety space around the body is particularly important in the presence of angry individuals who might be potentially harmful (Horstmann, 2003; Graziano and Cooke, 2006; Kennedy et al., 2009; Seidel et al., 2010; Iachini et al., 2015a). Supporting this view, previous work on the role of the stimuli valence has revealed that the presence of a dangerous object near the body produces shrinkage of the peripersonal space (Coello et al., 2012). Furthermore, Ruggiero et al. (2016) reported an increase of the peripersonal space when an angry avatar was approaching a participant in a virtual reality display. Both results are compatible with a peripersonal space representing a multimodal interface to interact safely with the physical and social environment (de Vignemont and Iannetti, 2015; Coello and Iachini, 2016b). In accordance with this view, unexpected invasion of peripersonal space may produce intense discomfort and anxiety (Horowitz et al., 1964; Hayduk, 1978; Lloyd, 2009). Furthermore, high trait anxiety is usually associated with an extended peripersonal space (Iachini et al., 2015b). In the present study, the protective role of peripersonal space is also highlighted by the observation that the PLDs located in the participants’ peripersonal space modulate the EDA, confirming the established link between threat and associated physiological response. In accordance with this, the more the participants rated stimuli as arousing, the more their physiological responses increased when the PLDs were in their peripersonal space (Sequeira et al., 2009; Bach et al., 2010). These results confirm thus the safety role of the peripersonal space and show how threatening stimuli have an impact on the physiological activity (McBride et al., 1965; Coello et al., 2012; Ferri et al., 2015; Rossetti et al., 2015; Szpak et al., 2015; Ruggiero et al., 2016).

As regards reachability judgments, the participants judged, as expected, almost all PLDs in peripersonal space as reachable (94.4%) and almost all PLDs in extrapersonal space as unreachable (99.10%). Concerning the PLDs located at the boundary of peripersonal space, the participants judged them as unreachable in 94.46% of the cases. This bias toward unreachability for stimuli located at the boundary of peripersonal space could be explained by the fact that the latter was determined in a pilot study using approaching stimuli. Previous studies have indeed shown that peripersonal space increased when a confederate approached a passive participant, in comparison to a situation where the participant was moving toward the confederate (Iachini et al., 2014; Ruggiero et al., 2016). The fact that the boundary of peripersonal space was specified in our study on the basis of approaching PLDs could explain the prevalence of unreachable responses when judging afterward the reachability of stationary PLDs.

With respect to the *interpersonal comfort distance*, the minimum distance was on average 30 cm (inter-shoulder distance), which is in agreement with previous studies (e.g., 32 cm in Quesque et al., 2017). We found that the minimum comfort distance increased with PLDs carrying angry facial expressions in comparison to PLDs with neutral ones (34%) and in comparison to PLDs with happy facial expressions (39%). The present data confirm the effect of valence of facial expressions on comfortable interpersonal distances (Lockard et al., 1977; Ruggiero et al., 2016). Facial expressions rated as negative (e.g., angry facial expressions) led to an increase of the comfortable interpersonal distance in comparison to those rated more positively (neutral and happy facial expressions). We also found that the more facial expressions were rated as arousing by individuals, the more the minimum comfort distance increased and that this relation was modulated by the valence evaluation of the same stimuli. The increase of minimum comfort distance in relation to the increase of arousal was indeed lower when the valence was rated positively. These findings corroborate the previous observation that spatial distance enlarges in the presence of angry faces compared to neutral and happy faces, with no difference between the last two (Ruggiero et al., 2016). However, the present study went further by demonstrating that this enlargement was also associated with the subjective evaluation of the faces (including both valence and arousal).

Surprisingly, neither the participants’ nor the PLDs’ gender was found to modulate the minimum comfort distance in the social interaction task, which contrasts to what was reported in previous research (e.g., McBride et al., 1965; Iachini et al., 2016). For instance, Iachini et al. (2016) described an increase of the minimum comfort distance from male virtual confederates in comparison to female ones. The main findings were that peripersonal space and interpersonal distances shrank with humans as compared to objects (Iachini et al., 2014), and both spaces were affected by age and gender, i.e., decreased with children and females as compared to adult males, thus reflecting, respectively, affiliative and attraction mechanisms (Iachini et al., 2016; see also Argyle and Dean, 1965; Aiello, 1987; Uzzell and Horne, 2006). The different effect of gender on interpersonal social space observed in these studies and the present one could be due to the importance of facial expressions, which may have prevented or reduced the effect of gender (see also Ruggiero et al., 2016). Although facial expressions and gait were gendered, the emotions displayed might capture most of the attention available while putting aside less relevant features such as gender.

Another important point raised by the present study concerns the relation between the physiological response associated with PLDs in the participants’ peripersonal space and the minimum comfort distance accepted with the same stimuli. When considering PLDs with angry and neutral facial expressions (i.e., the ones statistically different in the two tasks), we found a significant relation between the change of the EDA (reachability judgment task) and the change of the preferred social distance (comfort interpersonal distance judgment task), associated with the different valence of the facial expressions. We also observed that the more the physiological response increased in the presence of a negative facial expression, the more the interpersonal distance of comfort widened. Precisely, a gain of 0.01 μS for the phasic activity for stimuli presented in the peripersonal space corresponded to an increase of the comfort distance of 5.14 cm. Information regarding the emotional state of a confederate in a social context would trigger physiological automatic response likely to help adapting distance to the confederate in order to feel safe. It is worth noting that EDA was acquired during the reachability judgment task only and not also during the comfort interpersonal distance judgment task in order to avoid any habituation effect of the emotional stimuli on EDA, but which represents a limitation of the present study. Another extension of the present work would be to compare these data to the postural stability of participants while threatening stimuli are approaching them. This might indeed inform us about the implicit behavioral withdrawal strategy adopted along with the physiological responses. An additional interesting aspect would be to manipulate the characteristics of the PLDs in order to study whether other characteristics of the human-like stimuli (size, status, previous experience…) are taken into account to specify the spatial component of social interactions.

Taken together, these results confirm the protective role of peripersonal-action space and support its role in the adjustment of interpersonal comfort distances for appropriate social interactions (Iachini et al., 2014; Coello and Iachini, 2016a; Ruggiero et al., 2016; Quesque et al., 2017). The increase of the physiological response to PLDs with angry faces may represent an automatic avoidance reaction to the violation of the near body space, as a consequence of arousal regulation and the necessity to ensure a stable self-protection (Dosey and Meisels, 1969; Hayduk, 1983; Siegman and Feldstein, 2014). The strong physiological response in the presence of angry faces is consistent with neurofunctional and behavioral studies showing that negative stimuli yield stronger body response than positive stimuli (Öhman, 1987; Cacioppo et al., 1993; de Gelder et al., 1999; Strack and Deutsch, 2004; Vuilleumier and Pourtois, 2007; van Dantzig et al., 2008; Cole et al., 2013). Thus, the proximity of a threatening confederate obviously leads to avoidance mechanisms in the form of an increase of the social distance, with the consequence that non-appropriate social distance leads to physiological warning signal inducing defense behavior (Lockard et al., 1977; Evans and Wener, 2007; Kennedy et al., 2009; Ruggiero et al., 2016). In contrast, positive elements such as happy facial expressions might foster social interactions (Lockard et al., 1977; Cole et al., 2013; Ruggiero et al., 2016).

## Conclusion

The present study showed that both peripersonal-action space and interpersonal-social space are similarly sensitive to the emotional meaning of stimuli, which suggests that they may rely on common mechanisms in relation to the motor action system. It also brings new information regarding the emotional coding of threat in terms of distances and how safety can be quantified physiologically and spatially.

## Author Contributions

AC, GR, TI, and YC conceived and planned the experiments and contributed to writing the manuscript and interpretation of the results. AC, YC, and LO carried out the experiments and analyzed the data.

## Conflict of Interest Statement

The authors declare that the research was conducted in the absence of any commercial or financial relationships that could be construed as a potential conflict of interest.
